# Sex differences in the disposition of cannabidiol and its metabolites in mice

**DOI:** 10.1186/s42238-026-00427-7

**Published:** 2026-04-02

**Authors:** Margaret E. Olawale, Mrunmayi D. Lad, Mmesoma C. Anyachebelu, Shaman Luo, Philip Lazarus

**Affiliations:** Division of Molecular Biosciences, Department of Pharmaceutical Sciences, School of Pharmacy and Pharmaceutical Sciences, University at Buffalo, Buffalo, NY 14214 USA

**Keywords:** Cannabidiol, 7-hydroxy-CBD, 7-carboxy-CBD, Pharmacokinetics, Sex differences, Tissue distribution, UPLC-MS/MS

## Abstract

**Background:**

While cannabidiol (CBD) is widely used globally as a therapeutic agent, sex as a biological variable remains underexplored in determining its metabolism and overall pharmacokinetic patterns. The present study systematically evaluated sex differences in the pharmacokinetics of CBD and its major metabolites, 7-hydroxy-CBD (7-OH-CBD) and 7-carboxy-CBD (7-COOH-CBD), in a mouse model.

**Methods:**

Male and female C57BL/6J mice received an intraperitoneal dose of CBD (120 mg/kg) and plasma concentrations of CBD and its metabolites were quantified by UPLC–MS/MS. Pharmacokinetic parameters were derived using non-compartmental analysis and compared between sexes.

**Results:**

Females exhibited significantly higher early exposure to CBD, with a ~ 1.5-fold higher $\:{C}_{\mathrm{m}\mathrm{a}\mathrm{x}}\:$than male mice (*p* = 0.03). The apparent clearance (CL/F) and ultimate total systemic exposure$\:{\:AUC}_{0-\mathrm{I}\mathrm{N}\mathrm{F}}$ were comparable between sexes. In contrast, male mice demonstrated a markedly larger apparent volume of distribution (Vz/F; ~2.2-fold increase, *p* = 0.02) and consequently a longer terminal half-life (t_1/2_; ~2.2-fold increase, *p* = 0.04), indicating greater tissue sequestration. Both metabolites were significantly higher in female vs. male mice (7-OH-CBD $\:{C}_{\mathrm{m}\mathrm{a}\mathrm{x}}\:$~1.6-fold, *p* = 0.03; 7-COOH-CBD $\:{C}_{\mathrm{m}\mathrm{a}\mathrm{x}}$ ~1.7-fold, *p* = 0.03). The $\:{AUC}_{0-\mathrm{I}\mathrm{N}\mathrm{F}}$ tended to be higher for 7-OH-CBD in female mice but was not significant (~ 1.4-fold, *p* = 0.11), whereas the 7-COOH-CBD $\:{AUC}_{0-24\mathrm{h}}$ was significantly greater in females (~ 1.8-fold, *p* = 0.04). Male mice displayed substantially longer terminal half-lives for both metabolites (7-OH-CBD ~2.4-fold, *p* = 0.0051; 7-COOH-CBD ~3.7-fold, *p* = 0.02).

**Conclusions:**

These results demonstrate that sex is a critical determinant of CBD pharmacokinetics and highlight the need for sex-informed dosing considerations in both preclinical study design and potentially for future clinical applications of CBD.

**Supplementary Information:**

The online version contains supplementary material available at 10.1186/s42238-026-00427-7.

## Background

Cannabidiol (CBD) is the second most abundant phytocannabinoid derived from the cannabis sativa plant after Δ9-tetrahydrocannabinol (THC) (Premoli et al. 2019, Shahbazi et al. 2020, Mechoulam et al. 2007). Unlike THC, CBD is generally considered non-psychoactive, although some reports suggest it may exert mild central nervous system (CNS) effects (Stella 2023, Martínez et al. 2020, Oberbarnscheidt and Miller 2020). CBD interacts with the endocannabinoid system but has a low affinity for CB1 receptors, which are primarily located in the CNS and mediate the psychoactive effects of THC (Shahbazi et al. 2020, Zou and Kumar 2018). Additionally, CBD binds to CB2 receptors which are predominantly found in peripheral tissues including cells of immunity, where it may act through low affinity binding, allosteric modulation, or functional antagonism to influence immune and inflammatory processes (Zou and Kumar 2018, Peng et al. 2022). Other receptors that CBD has been reported to interact with include the G protein–coupled receptor 55 (GPR55), the serotonin 5-HT1A receptor, and the transient receptor potential vanilloid type 1 (TRPV1) channel, contributing to its proposed anticancer, analgesic, anxiolytic, addiction treatment and anti-inflammatory effects (Schouten et al. 2024, Miao et al. 2025, Almeida and Devi 2020, Filipiuc et al. 2021).

The use of CBD has increased rapidly in recent years, particularly following the passage of the 2018 United States (U.S.) Farm Bill, which legalized hemp-derived CBD products containing less than 0.3% of THC in all 50 states (Dickson et al. 2019, Park 2025). This regulatory shift significantly increased public accessibility and consumer interest, with U.S. retail CBD sales reported at approximately $5.3 billion in 2021 and $4.17 billion in 2022 (Park 2025, Bhamra et al. 2021, CBD ), with sales projected to reach $4.23 billion in 2026 (CBD ). Beyond legal availability, CBD has gained popularity due to widespread perception of its potential health benefits, which have contributed to its growing use among consumers (Nguyen et al. 2023). However, despite its extensive marketing and diverse claimed therapeutic benefits, robust scientific evidence supporting many of these uses remains limited (Krowartz et al. 2025, Chesney et al. 2020). Currently, the only U.S. Food and Drug Administration (FDA)-approved indication for CBD is as Epidiolex^®^, an oral solution indicated for the treatment of seizures associated with Lennox–Gastaut syndrome, Dravet syndrome, and tuberous sclerosis complex in children and adults (Jazz and Pharmaceuticals , Reddy 2023). Nonetheless, large numbers of individuals continue to self-administer over the counter (OTC) CBD formulations without medical supervision (Kaufmann et al. 2023). Given this growing use, a detailed understanding of CBD pharmacokinetics is essential to optimize its therapeutic potential, evaluate safety, and guide rational clinical application. Parameters such as bioavailability, maximum plasma concentration, clearance, and half-life are fundamental for establishing effective dosing regimens and predicting therapeutic outcomes. Without this knowledge, it is difficult to optimize treatment, anticipate drug–drug interactions, or evaluate potential toxicities (Millar et al. 2018, Martinez Naya et al. 2024).

**Fig. 1:**
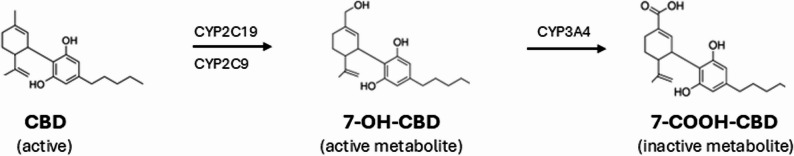
Metabolic pathway of CBD showing formation of active 7-OH-CBD and subsequent conversion to inactive 7-COOH-CBD. CYP, cytochrome P450

Although complex, CBD pharmacokinetics (PK) have been studied in both human and animal models; however, several major limitations remain (Millar et al. 2018, Martinez Naya et al. 2024, Wermer et al. 2025, Deiana et al. 2012). CBD exhibits low and variable oral bioavailability (estimated between 6 and 19%), largely due to poor solubility and extensive first-pass metabolism (Lacerda et al. 2025, Hossain et al. 2023). Following administration, CBD is metabolized in humans by cytochrome P450 enzymes in the liver, leading to the formation of both active and inactive metabolites (Fig. 1) (Bardhi et al. 2022). The active metabolite, 7-hydroxy-CBD (7-OH-CBD), is primarily formed by CYP2C19 and CYP2C9, while CYP3A4 contributes to the formation of 7-carboxy-CBD (7-COOH-CBD), the most abundant circulating metabolite but is considered pharmacologically inactive (Beers et al. 2021). In mice, CBD is predominantly metabolized by enzymes from the CYP2C and CYP3A families, which are considered functional counterparts to the human CYP2C and CYP3A isoforms and generate the same primary metabolites [7-OH-CBD and 7-COOH-CBD (Martin et al. 1977, Bornheim and Correia 1990)]. These metabolites, particularly 7-OH-CBD, may contribute significantly to CBD’s therapeutic and safety profile, but most studies have focused primarily on parent CBD, with limited systematic analysis of metabolite kinetics (Zhang et al. 2024, Ujváry and Hanuš 2016). Furthermore, while a growing body of literature has described CBD PK under various dosing routes, few studies have rigorously evaluated sex as a biological variable, despite its well-recognized influence on drug metabolism (Sallam et al. 2023, MacNair et al. 2024).

Sex is a recognized determinant of drug disposition, reflecting differences in enzyme expression/activity, protein binding, and body composition in both rodents and humans (Waxman and Holloway 2009, Aljohmani and Yildiz 2025). However, evidence for sex as a biological variable in CBD PK is limited and mixed. In a repeated oral CBD study in healthy adults, sex differences in CBD’s area under the plasma concentration-time curve (AUC) and maximum plasma concentration $\:\left({C}_{\mathrm{max}}\right)$, were limited, but the relative exposure of metabolites compared to parent drug increased more in females across days (MacNair et al. 2024). While this suggests an altered exposure profile by sex, rigorous preclinical studies that quantified parent and metabolite PK under controlled conditions and that mechanistically disentangled the role of distribution from elimination are scarce. Most published preclinical studies either pooled data from both sexes or restricted analysis to males, overlooking a potentially important determinant of CBD exposure.

To address this gap in knowledge, we hypothesized that there will be significant sex-dependent differences in the PK of high-dose CBD and its metabolites in mice. Specifically, the present study aimed to characterize sex-dependent plasma concentration-time profiles of CBD and its major metabolites in mice following high-dose intraperitoneal (IP) administration, to compare key PK parameters between the two sexes and provide a foundation for future studies exploring the biological and clinical implications of sex-based differences in CBD disposition.

## Materials and methods

### Animals and housing

All animal ordering, housing, and study procedures were conducted in accordance with the Institutional Animal Care and Use Committee (IACUC) guidelines at the University at Buffalo under protocol number PROTO202400076. 12 C57BL/6J mice (6 males and 6 females, approximately 4-week-old, ~ 20 g in weight) were sourced from Jackson Laboratory (Bar Harbor, ME, USA) and acclimated for 5 days in a conventional rodent room. Male and female mice were housed separately, in groups of three per cage with bedding, food, and water provided. Animals were monitored daily for signs of distress, including movement and grooming to ensure wellbeing throughout the acclimation and experimental periods.

### Drugs and chemicals

 Non-pharmaceutical grade cannabidiol (CBD) was purchased from GVB Biopharma (Portland, OR, USA), Cremophor EL from EMD Millipore Corp. (Burlington, MA, USA), ethanol from Decon Labs, Inc. (King of Prussia, PA, USA), and sterile saline 0.9% from Alkaline Scientific (Fort Lauderdale, FL. USA). CBD was prepared at a concentration of 120 mg per 5 mL in a mixture of cremophor EL: ethanol: saline vehicle (1:1:18, v/v/v) in accordance with manufacturer recommendations. CBD was first dissolved in 0.25 mL cremophor EL and 0.25 mL ethanol with heating at 60 °C, followed by the addition of 4.5 mL sterile saline. The resulting stock solution was aliquoted into 1.5 mL tubes, and stored at − 20 °C, and was thawed at room temperature immediately prior to use. Thawed aliquots were visually inspected to confirm the absence of precipitation before use.

For bioanalytical analysis, CBD reference standard solutions were prepared at a concentration of 1 mg/mL by dissolving CBD powder in methanol. Reference standards for 7-OH-CBD and 7-COOH-CBD, along with their corresponding deuterated internal standards (CBD-D_3_, 7-OH-CBD-D_3_, and 7-COOH-CBD-D_3_), were obtained from Sigma-Millipore (Burlington MA, USA). Organic solvents and mobile-phase modifiers used for ultra performance liquid chromatography-mass spectrometry (UPLC–MS/MS) analysis including acetonitrile and formic acid were all LC–MS grade. Acetonitrile, methanol, and formic acid were purchased from Thermo-Fisher Scientific (Waltham, MA, USA), and high-purity water (Milli-Q) was used for the preparation of all aqueous solutions.

### Experimental methods

Each mouse was weighed prior to dosing to calculate the injection volume, corresponding to a CBD dose of 120 mg/kg in 5 mL/kg (approximately 100 µL for a 20 g mouse). At time 0, CBD was administered via IP injection using a 25-gauge needle inserted at a 45° angle into the lower abdominal quadrant, while the mouse was gently restrained in a supine position. Injections were administered slowly to ensure consistent delivery and minimize local irritation. Following dosing, mice were monitored for movement, activity, and posture throughout the experiment, and for signs of distress like sedation, mild ataxia, and hypotension.

Blood samples (~ 12 µL each; total volume < 10% of the estimated blood circulating volume of ~ 1,600 µL) were collected from the submandibular vein at 5 min, 10 min, 15 min, 30 min, 45 min, 1 h, 2 h, 3 h, 6 h, 12 h, and 24 h post-injection. At the 24-hour time point, mice were euthanized by cardiac puncture followed by cervical dislocation as a secondary method. Plasma was separated by centrifugation at 2,500 × g for 10 min and stored at − 80 °C for quantification of CBD and its metabolites, 7-OH-CBD and 7-COOH-CBD, via UPLC-MS/MS.

### Bioanalytical assay (UPLC-MS/MS)

Standard curve solutions were prepared by spiking blank plasma with reference standards to yield concentrations ranging from 50 to 25,600 ng/mL. For both calibration standards and test plasma samples, protein precipitation was performed by combining plasma, internal standard solution (0.5 ppm mixture of all deuterated internal standards, prepared in acetonitrile), and cold acetonitrile in a ratio of 1:1:3. Specifically, 5 µL of plasma was mixed with 5 µL of internal standard and 15 µL of acetonitrile, followed by vortex mixing and centrifugation at 17,000 × g for 10 min at 4 °C. The resulting supernatant was collected to produce the final calibration standards, and the analysis was performed by injecting 5 µL of the supernatant into the UPLC-MS/MS.

The quantification of CBD, 7-OH-CBD, and 7-COOH-CBD, was performed using a Waters Acquity UPLC system coupled to a XEVO-TQSmicro mass spectrometer (Waters Corp, Milford, MA). Chromatographic separation was achieved using an ACQUITY UPLC BEH C18 Column (130Å, 1.7 μm, 2.1 mm X 50 mm) maintained at 40 °C. The mobile phase consisted of (A) water with 0.1% formic acid, and (B) acetonitrile with 0.1% formic acid, delivered at a flow rate of 0.3 mL/min with the following 8.5-min gradient. The gradient was initiated at 60% A and held for 2 min, then decreased linearly to 20% A at 3.5 min, further decreased linearly to 5% A at 7 min, held at 5% A from 7 to 7.5 min, returned to 80% A by 8.5 min, and then returned to initial conditions for the subsequent injection. Detection was performed in positive electrospray ionization mode using multiple reaction monitoring (MRM). Instrument settings were as follows: capillary voltage, 0.6 kV; cone voltage, 20 V. Collision energies were set at 20 eV for CBD, 10 eV for 7-OH-CBD, and 15 eV for 7-COOH-CBD. The MRM transitions monitored were m/z 315.2/193.1 for CBD, 318.2/196.2 for CBD-D_3_, 331.2/313.3 for 7-OH-CBD, 334.2/316.1 for 7-OH-CBD-D_3_, 345.1/299.3 for 7-COOH-CBD and 348.2/302.2 for 7-COOH-CBD-D_3_.

### UPLC-MS/MS method qualification

Selectivity was assessed using blank plasma obtained from six individual mice processed with internal standard. No endogenous peaks exceeding 20% of the lower limit of quantification (LLOQ) response for CBD, 7-OH-CBD, or 7-COOH-CBD were observed at their respective retention times, and no endogenous responses exceeded 5% of the internal standard signal (Supplementary Fig. 1), meeting FDA and EMA acceptance criteria (Health UDo, Services 2003, Guideline 2022). In addition, mouse plasma spiked with 400 ng/mL of the analytes, along with their corresponding deuterated internal standards, demonstrated well-resolved peaks at the expected retention times, confirming analyte identity and the absence of matrix interference (Supplementary Fig. 2).

The assay showed sufficient sensitivity for all analytes. LLOQs were initially determined based on signal-to-noise ratios ≥ 10 from calibration curves and subsequently confirmed by five replicate measurements. Based on five replicate measurements CBD exhibited an LLOQ of 25 ng/mL, while 7-OH-CBD and 7-COOH-CBD met LLOQ criteria at 50 ng/mL, demonstrating accuracy within 80–120% of nominal concentration and a precision (CV%) ≤ 20% (Supplementary Table 1). Importantly, all plasma concentrations measured in test samples across the 24-hour sampling period were above the established LLOQs, confirming the integrity of the data used for pharmacokinetic analysis. This method therefore met FDA and EMA criteria for selectivity and sensitivity (Health UDo, Services 2003, Guideline 2022). Linearity across the calibration range (50–25,600 ng/mL) was excellent, with correlation coefficients (R²) exceeding 0.99 for all three analytes using a weighted (1/x) linear regression model.

Additionally, intra-day accuracy and precision were evaluated using five replicate quality control (QC) samples per concentration level on a single day, while inter-day accuracy and precision were assessed using five replicate QC samples per concentration level analyzed across three separate days (total of 15 measurements per concentration). QC samples were independently prepared by spiking blank mouse plasma with reference standards at low (200 ng/mL), medium (6,400 ng/mL), and high (12,800 ng/mL) concentrations and were processed in the same manner as the study sample. All QC samples met FDA acceptance criteria, with accuracy within 85–115% of nominal values and precision (CV%) ≤ 15% for both intra- and inter-day measurements (Supplementary Table 2) (Health UDo, Services 2003).

### Data analysis

All PK data are presented as the mean ± SD from six animals per group. PK parameters were derived using Non-Compartmental Analysis (NCA) in Phoenix WinNonlin 8.6 [Certara USA, Inc., (Princeton, NJ, USA)]. For CBD and 7-OH-CBD, the area under the plasma concentration–time curve from time zero to infinity ($\:{AUC}_{0-\mathrm{I}\mathrm{N}\mathrm{F}}$) was calculated since both compounds exhibited a well-defined terminal elimination phase. In contrast, for 7-COOH-CBD, the elimination phase was incomplete within the 24-hour sampling period; therefore, exposure was reported as area under the plasma concentration–time curve from time zero to 24 h$\:\:\left({AUC}_{0-24\mathrm{h}}\right)$, representing the observed exposure over the measurable time course. Other measured parameters included maximum plasma concentration ($\:{C}_{\mathrm{max}})$, time to reach maximum plasma concentration ($\:{t}_{\mathrm{max}}),\:$apparent clearance (CL/F), and apparent volume of distribution (Vz/F), and terminal half-life (t_1/2_).

Statistical analyses were performed using GraphPad Prism (version 10). Plasma concentration–time data were analyzed using a two-way analysis of variance (ANOVA) with sex and time as factors, followed by post-hoc comparisons using Bonferroni’s multiple comparisons test to assess differences between males and females at individual time points and differences were considered significantly at *p* ≤ 0.05. Comparisons of all derived PK parameters between male and female groups were performed using unpaired two-tailed t-tests, with statistical significance set at *p* ≤ 0.05.

## Results

### Plasma concentration-time profile of CBD and its metabolites

**Fig. 2:**
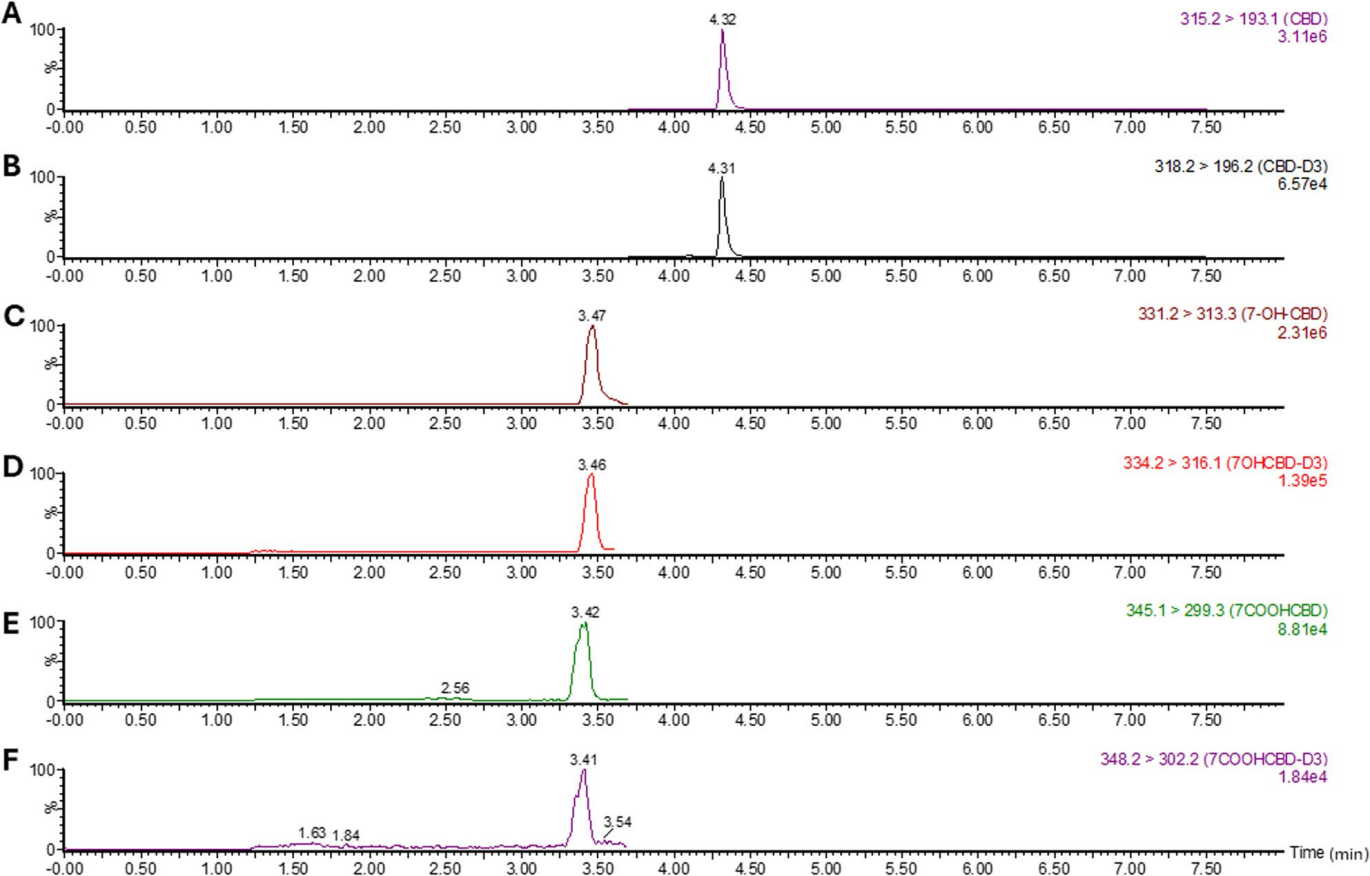
Representative chromatograms of CBD and its metabolites along with their deuterated internal standards in plasma samples obtained after CBD administration. **A** CBD; **B** CBD-D3; **C** 7-OH-CBD; **D** 7-OH-CBD-D3; **E** 7-COOH-CBD; and **F** 7-COOH-CBD-D3. Data shown are from a representative C57BL/6J mouse plasma sample collected after intraperitoneal CBD administration, acquired in ESI+ mode following protein precipitation and reversed-phase separation; retention times and mass transition are annotated above each peak

Representative UPLC–MS/MS chromatograms confirming the detection of CBD and its metabolites (7-OH-CBD and 7-COOH-CBD) and their corresponding deuterated internal standard, obtained from plasma sample collected from CBD-treated mice are shown in (Fig. 2). Each analyte was well resolved with distinct retention times and no interference from blank plasma, confirming the specificity of the analytical method.

**Fig. 3:**
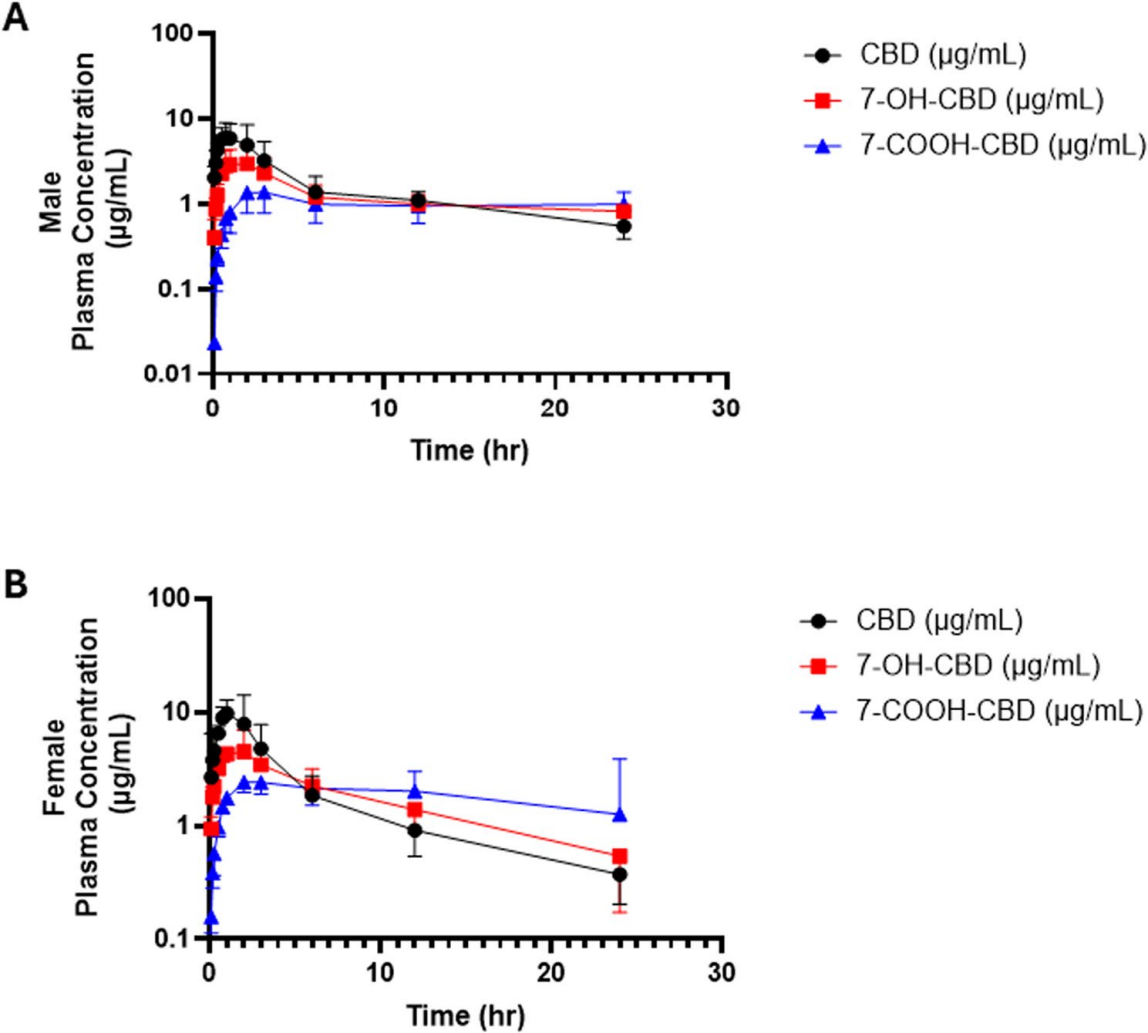
Plasma concentration–time profiles of CBD and its metabolites, 7-OH-CBD and 7-COOH-CBD, in male (panel **A**) and female (panel **B**) C57BL/6J mice following a single 120 mg/kg IP dose of CBD. Data are shown as mean ± SD (*n* = 6 per sex)

Following a single IP administration of CBD at 120 mg/kg to both male and female C57BL/6J mice, CBD was rapidly detected in plasma and was subsequently metabolized to 7-OH-CBD and 7-COOH-CBD for both male and female mice (Fig. 3). Plasma concentrations of CBD increased sharply reaching peak plasma concentrations (C_max_) within the first hour post-dose, indicating rapid systemic absorption, and then declined in a multiphasic manner consistent with distribution and elimination processes. Formation of 7-OH-CBD occurred shortly after CBD appearance, followed by a delayed rise in 7-COOH-CBD, consistent with sequential oxidation and carboxylation of CBD. Both metabolites persisted longer in circulation than the parent compound, a finding consistent with their slower systemic clearance. The overall temporal patterns confirm that CBD undergoes extensive in vivo metabolism and that all three analytes exhibit measurable systemic exposure over the 24-hour sampling period.

### Sex differences in CBD plasma concentrations and PK

**Fig. 4:**
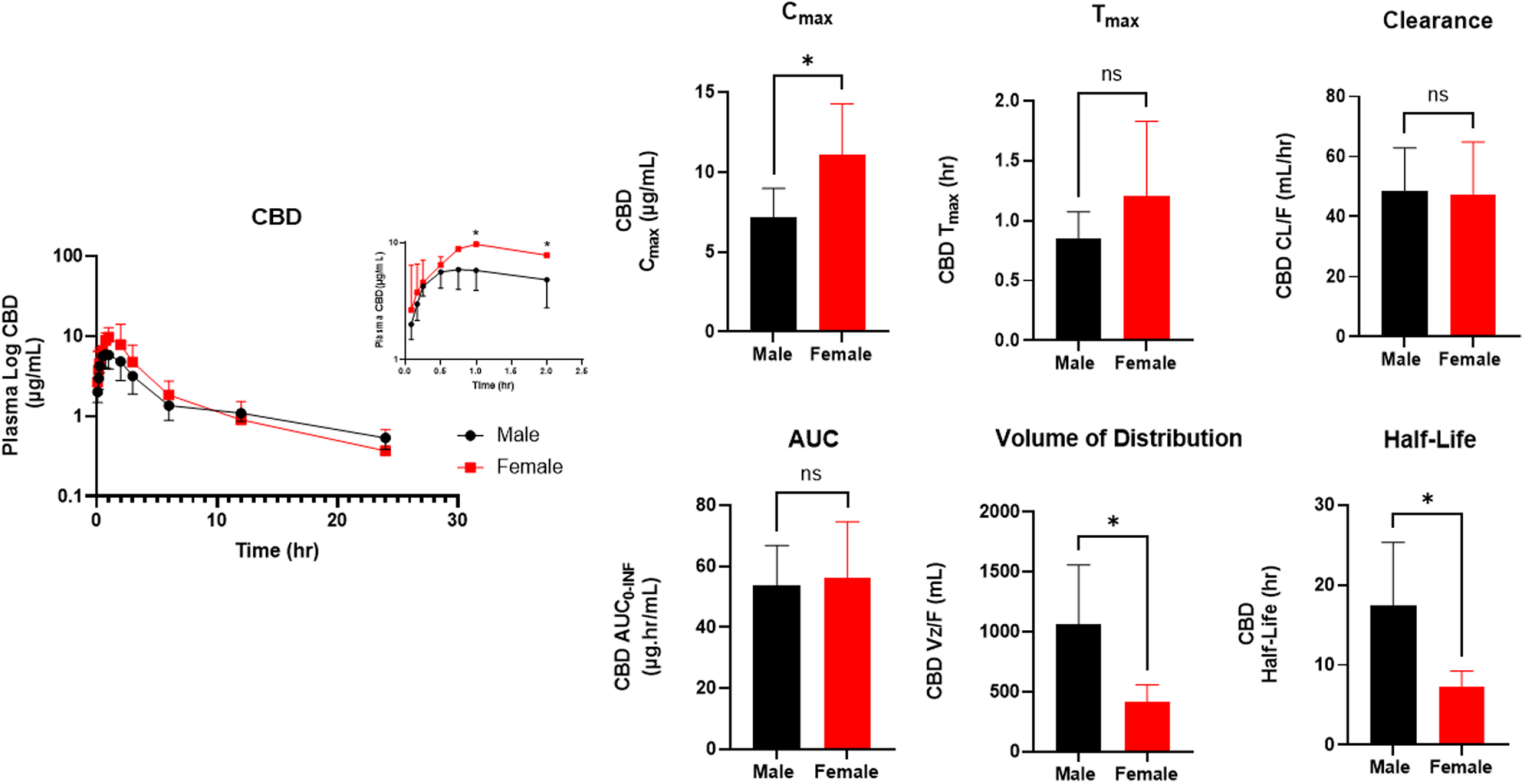
Plasma concentration–time profile and PK parameters of CBD following a single 120 mg/kg IP dose in male and female C57BL/6J mice. Shown are plasma concentration–time curves and NCA parameters ( and $\mathrm{t}_{1/2}$) The inset highlights the plasma CBD concentration–time profile at earlier time points (up to 2 h). Bars are presented as mean ± SD (*n* = 6 per sex). *P < 0.05; ns = not significant

To examine whether CBD disposition differs between sexes, plasma concentration–time curves were compared between male and female mice following a single 120 mg/kg IP dose of CBD (Fig. 4). Both groups exhibited rapid absorption, with plasma concentrations peaking within the first hour post-dose, followed by a multiphasic decline over 24 h. Female mice showed higher plasma CBD concentrations than males during the early absorption phase (approximately 1–2 h post-dose) before redistribution and elimination occurred, suggesting transiently greater systemic exposure at the onset of distribution. A two-way ANOVA revealed a significant main effect of time (F _(11, 109)_ = 33.0, *p* < 0.0001) and a significant interaction between sex and time (F _(11, 109)_ = 1.9, *p* = 0.04), indicating that CBD concentrations over time differed between sexes. Although the main effect of sex was not significant (F _(1, 10)_ = 4.2, *p* = 0.068), post hoc Bonferroni comparisons showed that female mice had significantly higher plasma CBD levels than males at 1 h and 2 h post-dose (*p* = 0.013 and 0.049, respectively; Fig. 4).

Based on the observed sex-related differences in the plasma concentration–time curves, PK parameters derived from non-compartmental analysis (NCA) were compared between male and female mice (Table 1 and Fig. 4). Female mice exhibited a significantly higher $\:{C}_{\mathrm{max}}$ than males (7.2 ± 1.8 µg/mL vs. 11.1 ± 3.2 µg/mL, *p* = 0.03), with comparable $\:{t}_{\mathrm{max}}$ values (males: 0.74 ± 0.34 h; females: 1.2 ± 0.62 h, *p* = 0.20). This is consistent with their elevated CBD levels during the early absorption phase identified in the two-way ANOVA. Despite this initial difference in $\:{C}_{\mathrm{max}},\:$the CL/F was similar between groups (males: 48.4 ± 14.5 mL/h; females: 47.4 ± 17.3 mL/h, *p* = 0.92), and total systemic exposure as measured by $\:{AUC}_{0-\mathrm{I}\mathrm{N}\mathrm{F}}$, did not differ significantly between sexes (53.6 ± 13.2 µg·h/mL vs. 55.9 ± 18.7 µg·h/mL, *p* = 0.81). Male mice showed a markedly larger apparent volume of distribution (Vz/F) (1060 ± 500 mL vs. 480 ± 180 mL, *p* = 0.02) and a longer terminal half-life (t_1/2_): (15.7 ± 8.2 h vs. 7.2 ± 2.0 h, *p* = 0.04) compared with females.

**Table 1:** PK parameters of CBD

Parameters	Unit	Male	Female	*P*-value^a^
$\:{\boldsymbol{C}}_{\mathrm{max}}$	µg/mL	7.2 ± 1.8	11.1 ± 3.2	**0.03**
$\:{\boldsymbol{T}}_{\mathrm{max}}$	h	0.74 ± 0.34	1.2 ± 0.62	0.20
$\:{\boldsymbol{A}\boldsymbol{U}\boldsymbol{C}}_{0-\mathbf{I}\mathbf{N}\mathbf{F}}$	µg·h/mL	53.6 ± 13.2	55.9 ± 18.7	0.81
Vz/F	mL	1060 ± 500	480 ± 180	**0.02**
$\:{\boldsymbol{T}}_{1/2}$	h	15.7 ± 8.2	7.2 ± 2.0	**0.04**
CL/F	mL/h	48.4 ± 14.5	47.5 ± 17.3	0.92

Data are presented as mean ± SD (*n* = 6 per sex)

^a^ Significant *P*-values are in bold

### Sex differences in CBD metabolites plasma concentrations and PK

**Fig. 5:**
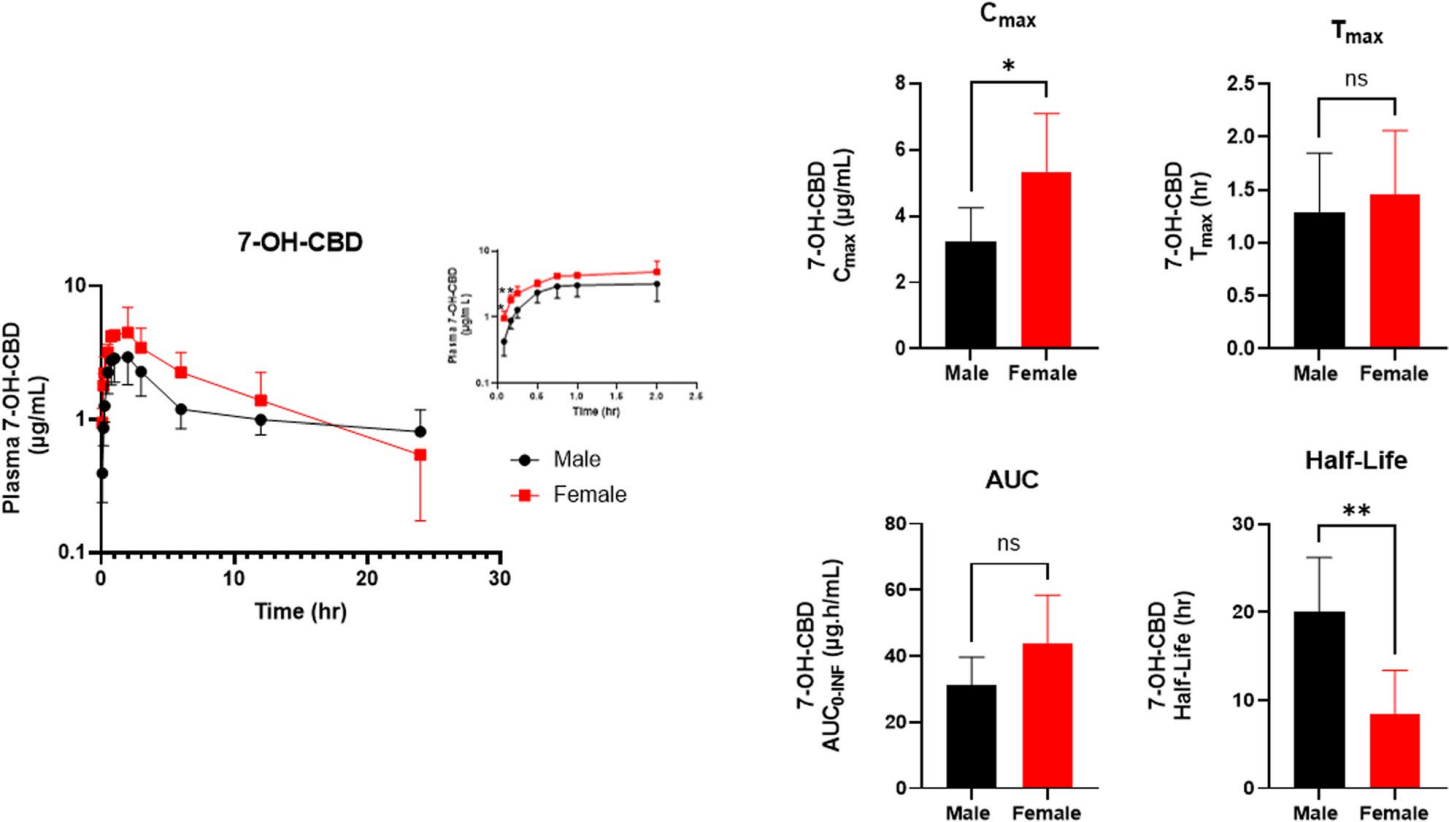
Plasma concentration–time profile and PK parameters of 7-OH-CBD following a single 120 mg/kg IP dose of CBD in male and female C57BL/6J mice. Shown are plasma concentration–time curves and NCA parameters ($\:{C}_{\mathrm{max}}$,$\:{\:\:t}_{\mathrm{max}}$, $\:{AUC}_{0-\mathrm{I}\mathrm{N}\mathrm{F}}$, and t_1/2_). The inset highlights the plasma 7-OH-CBD concentration–time profile at earlier time points (up to 2 h). Bars are presented as mean ± SD (*n* = 6 per sex). *P < 0.05; **P < 0.01; ns = not significant

After CBD dosing, 7-OH-CBD appeared rapidly in plasma for both sexes and peaked within the first hour (Fig. 5). A two-way ANOVA showed a strong main effect of time (F _(1.9, 19.0)_ = 46.2, *p* < 0.0001) and a significant main effect of sex (F _(1,10)_ = 9.4, *p* = 0.01), whereas the time and sex interaction was not significant **(**F _(1.92, 18.98_) = 1.8, *p* = 0.19). Bonferroni post-hoc tests indicated significant higher 7-OH-CBD concentrations in females at the early absorption time points (0.083 h and 0.16 h; *p* = 0.038 and 0.0056, respectively), with no differences thereafter (Fig. 5). Together, these findings indicate a brief early-phase elevation of 7-OH-CBD in females, consistent with the greater early formation from CBD, while the subsequent time course is otherwise comparable between sexes.

To further evaluate the sex-dependent differences observed in plasma 7-OH-CBD concentrations, NCA was performed comparing male and female mice (Table 2 and Fig. 5). Consistent with the early time-course differences, females showed a higher 7-OH-CBD $\:{C}_{\mathrm{max}}\:$than males (male: 3.3 ± 1.0 µg/mL vs. female: 5.3 ± 1.75 µg/mL, *p* = 0.03), occurring at similar $\:{t}_{\mathrm{max}}$ values (male: 1.3 ± 0.56 h; female: 1.5 ± 0.60 h, *p* = 0.62). Additionally, $\:{AUC}_{0-\mathrm{I}\mathrm{N}\mathrm{F}}\:$was numerically higher in females but not statistically significant (males: 31.1 ± 8.6 µg·h/mL vs. females: 43.6 ± 14.8 µg·h/mL, *p =* 0.11). Finally, male mice exhibited a longer terminal half-life for 7-OH-CBD than females (males: 20.1 ± 6.1 h vs. females: 8.4 ± 5.0 h, *p* = 0.0051).

**Table 2:** PK parameters of 7-OH-CBD

Parameter	Unit	Males	Females	*P*-value^a^
$\:{\boldsymbol{C}}_{\mathrm{max}}$	µg/mL	3.3 ± 1.0	5.3 ± 1.78	**0.03**
$\:{\boldsymbol{T}}_{\mathrm{max}}$	h	1.3 ± 0.56	1.5 ± 0.60	0.62
$\:{\boldsymbol{A}\boldsymbol{U}\boldsymbol{C}}_{0-\mathbf{I}\mathbf{N}\mathbf{F}}$	µg·h/mL	31.1 ± 8.6	43.6 ± 14.8	0.11
$\:{\boldsymbol{T}}_{1/2}$	h	20.1 ± 6.1	8.4 ± 5.0	**0.0051**

Data are presented as mean ± SD (*n* = 6 per sex)

^a^ Significant *P*-values are in bold

**Fig. 6:**
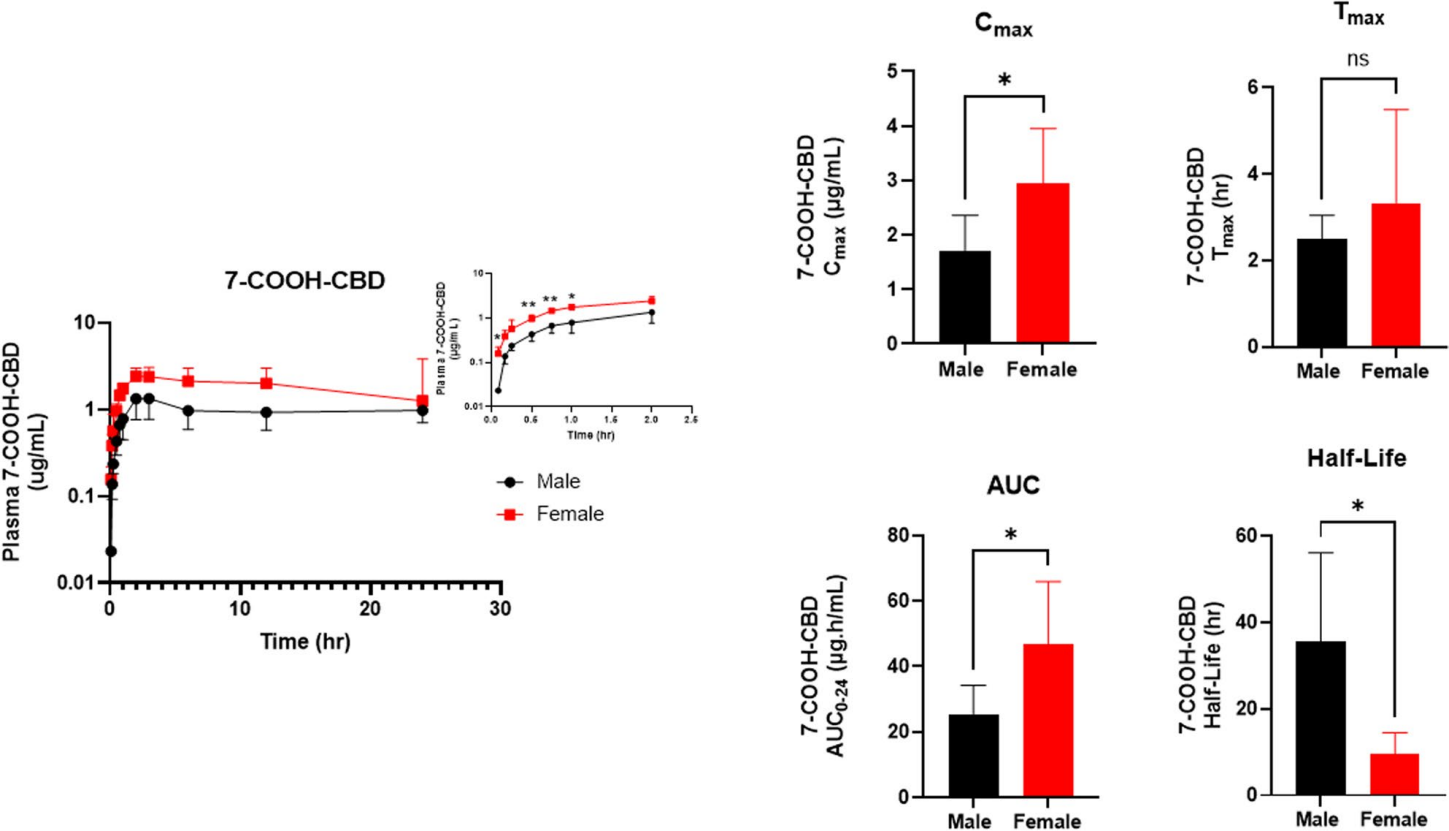
Plasma concentration–time profile and PK parameters of 7-COOH-CBD following a single 120 mg/kg IP dose of CBD in male and female C57BL/6J mice. Shown are plasma concentration–time curves and NCA parameters ($\:{C}_{\mathrm{max}}$,$\:{\:\:t}_{\mathrm{max}}$, $\:{AUC}_{0-24}$and t_1/2_). The inset highlights the plasma 7-COOH-CBD concentration–time profile at earlier time points (up to 2 h). Bars are presented as mean ± SD (*n* = 6 per sex). *P < 0.05; **P < 0.01; ns = not significant

As the secondary metabolite, 7-COOH-CBD concentrations rose rapidly following the initial appearance of 7-OH-CBD, maintaining readily detectable levels across the entire 24 h sampling (Fig. 6). Two-way ANOVA showed a strong main effect of time (F _(2.53, 25.07)_ = 30.9, *p* < 0.0001) and a significant main effect of sex (F _(1,10)_ = 17.8, *p* = 0.0018), whereas the time and sex interaction was not significant (F _(2.53, 25.07)_ = 2.3, *p* = 0.11). Bonferroni post-hoc comparisons indicated higher female concentrations during early absorption (0.083 h, 0.50 h, 0.75 h, and 1.00 h; *p* = 0.031, 0.0046, 0.0026, and 0.010, respectively), with no differences at later times (Fig. 6). Overall, while 7-COOH-CBD levels were sustained across the 24 h sampling interval in both sexes, females exhibited a greater transient early elevation.

NCA was used to evaluate the sex differences in plasma drug 7-COOH-CBD concentrations between male and female mice (Table 3 and Fig. 6). Female mice reached a higher 7-COOH-CBD $\:{C}_{\mathrm{max}}$ than males (male: 1.7 ± 0.65 µg/mL vs. females: 3.0 ± 1.0 µg/mL, *p* = 0.03). $\:{T}_{\mathrm{max}}\:$occurred at 2.5 ± 0.55 h for male mice vs. 3.3 ± 2.2 h for female mice (*p* = 0.39). Overall exposure was greater in females, with$\:\:{AUC}_{0-24\mathrm{h}}$ significantly exceeding male values (males: 25.5 ± 8.8 µg·h/mL vs. females: 43.8 ± 19.2 µg·h/mL, *p* = 0.04), while the terminal phase was prolonged in males, with a longer t_1/2_ (35.7 ± 20.4 h) compared with females (9.7 ± 4.9 h) (*p* = 0.02). These NCA results align with the concentration–time profiles, indicating higher peak and cumulative 7-COOH-CBD levels in females over the 24 h interval.

**Table 3:** PK parameters of 7-COOH-CBD

Parameters	Unit	Male	Female	*P*-value^a^
$\:{\boldsymbol{C}}_{\mathrm{max}}$	µg/mL	1.7 ± 0.65	3.0 ± 1.0	**0.03**
$\:{\boldsymbol{T}}_{\mathrm{max}}$	h	2.5 ± 0.55	3.3 ± 2.2	0.39
$\:{\boldsymbol{A}\boldsymbol{U}\boldsymbol{C}}_{0-24\boldsymbol{h}}$	µg·h/mL	25.5 ± 8.8	43.8 ± 19.2	**0.04**
$\:{\boldsymbol{T}}_{1/2}$	h	35.8 ± 20.4	9.7 ± 4.9	**0.02**

Data are presented as mean ± SD (*n* = 6 per sex)

^a^ Significant *P*-values are in bold

## Discussion

The current study characterizes the sex-dependent pharmacokinetic profiles of CBD and its major metabolites, 7-OH-CBD and 7-COOH-CBD, in C57BL/6J mice. Significant differences in CBD metabolism were observed in male vs. female mice, characterized by higher peak concentrations in females and a significantly larger volume of distribution and longer half-life in males. In C57BL/6J mice given the same IP dose of CBD, sex influenced where and for how long CBD and its metabolites resided in the body rather than how fast it was eliminated. While female mice displayed higher early plasma concentrations of CBD (~ 1.5-fold higher $\:{C}_{\mathrm{max}}$), the apparent clearance and ultimately overall exposure (CL/F and $\:{AUC}_{0-\mathrm{I}\mathrm{N}\mathrm{F}}$) for CBD was similar between sexes. Males, by contrast, exhibited a larger apparent volume of distribution (V_Z_/F; ~2.2-fold higher) and a longer terminal t_1/2_ (~ 2.2-fold higher). Taken together, these findings point to distribution or redistribution rather than net elimination capacity as the principal driver of the sex effect on CBD kinetics.

These findings can be mechanistically interpreted by referring to fundamental PK relationships, which explicitly distinguish the roles of clearance and distribution. The total systemic exposure, denoted by AUC, is related to apparent clearance (CL/F) by the relationship:

$$\:AUC=\frac{Dose}{CL/F}$$

Since both the administered dose and the CL/F were comparable between sexes, it is expected that the total systemic exposure would not differ significantly between these groups, as was observed in the present studies. Furthermore, the elimination half-life (t_1/2_) is fundamentally defined by the relationship:

$$\:{t}_{1/2}=\frac{0.693\times\:Vz/F}{CL/F}$$

This equation demonstrates that half-life is directly proportional to the apparent volume of distribution (Vz/F) and inversely proportional to the apparent clearance (CL/F). Thus, an increase in distribution or decrease in clearance will prolong elimination half-life. Because CL/F is similar between sexes, the markedly longer $\:{t}_{1/2}$ observed in males is directly attributed to their significantly larger Vz/F. This confirms that the prolonged persistence of CBD in male plasma samples is not due to slower elimination from the body, but rather to extensive sequestration and delayed release from a large, peripheral tissue compartment.

The height of the peak$\:\:\left({C}_{\mathrm{m}\mathrm{a}\mathrm{x}}\right)$ reflects the balance between how quickly a drug enters the systemic circulation (absorption) and its extent of distribution and elimination. Qualitatively, a higher peak can result from faster absorption or less extensive distribution during the absorption phase. In the present study, $\:{AUC}_{0-\mathrm{I}\mathrm{N}\mathrm{F}}$, CL/F and $\:\:{t}_{\mathrm{max}}$ were comparable between sexes, with values similar to prior reports using the same dose and IP formulations of CBD in mice (Deiana et al. 2012), This indicates a similar extent and rate of absorption and clearance. Therefore, the simplest interpretation for the higher early plasma CBD concentration seen in females is due to reduced early distribution, yielding a higher $\:{C}_{\mathrm{max}}$.

The sex-dependent distribution and resulting systemic availability of CBD provide the framework for interpreting the kinetics of its metabolites, 7-OH-CBD and 7-COOH-CBD. For both metabolites, female mice showed significantly higher peaks (7-OH-CBD: 1.6-fold higher$\:\:{C}_{\mathrm{m}\mathrm{a}\mathrm{x}}$; 7-COOH-CBD: 1.7-fold higher $\:{C}_{\mathrm{m}\mathrm{a}\mathrm{x}}$). Although hepatic CYP3A isoforms are reported to be expressed at higher basal levels in female mice (Fashe et al. 2025, Sakuma et al. 2009) and could, in principle, influence metabolite formation, the absence of a sex difference in apparent CBD clearance (CL/F) indicates that differences in metabolic capacity are unlikely to account for the observed pharmacokinetic differences. This pattern is therefore best explained as a direct consequence of the parent drug kinetics: higher early CBD female plasma delivers more substrate to hepatic enzymes, driving greater early formation of 7-OH-CBD and subsequently greater overall exposure for both metabolites. The $\:{AUC}_{0-\mathrm{I}\mathrm{N}\mathrm{F}}$ of 7-OH-CBD was ~ 1.4 fold higher and the$\:{AUC}_{0-24\mathrm{h}}$ of 7-COOH-CBD was 1.8-fold higher in female mice as compared with male mice. Similar parent-driven metabolite peaks have been described for other cannabinoids [e.g., Δ⁹-tetrahydrocannabinol (THC)], where earlier/higher parent exposure in females is associated with corresponding earlier/higher metabolite exposure (Sallam et al. 2023, Wiley and Burston 2014).

Despite the female mice showing greater overall exposure to the CBD metabolites, the terminal phase of both metabolites follows the principle established for CBD: male mice exhibit significantly longer half-lives (7-OH-CBD: 2.4-fold higher $\:{t}_{1/2}$; 7-COOH-CBD: 3.7-fold higher $\:{t}_{1/2}$). Because these metabolites share similar structural backbone with CBD, their terminal phases likely mirror the parent drug’s disposition. This pattern strongly suggests that CBD has a propensity for extensive tissue sequestration in males, reflected by a larger apparent distribution volume, and that the terminal behavior of its hydroxylated and carboxylated metabolites is closely linked to this parent drug disposition. Thus, in males, the longer terminal half-life for the parent drug and its metabolites is likely redistribution-rate limited, governed by the slow release from the large peripheral tissue depot back into the systemic circulation.

These findings suggest that preclinical studies investigating CBD in epilepsy and other neurological or drug-related disorders should consider analyzing and reporting pharmacokinetic and pharmacodynamic outcomes by sex rather than pooling data across sexes, as this approach may enable more accurate inference and mechanistic interpretation. In practical terms, the present data demonstrate that sex differentiates the timing and distribution of CBD exposure when a similar dose is administered. Female mice reached higher early concentrations of CBD and its metabolites, whereas males retained the parent drug and both metabolites, particularly the inactive 7-COOH-CBD, for a significantly longer duration. This suggests distinct profiles even after a single administration.

The female exposure profile is front-loaded, characterized by a more rapid rise in systemic concentrations, which may increase sensitivity to peak-related effects in preclinical models. Hence, dosing strategies that reduce the rapid early rise in plasma concentrations may be considered in female mice. These include lowering initial dose and considering schedules or formulations that prevent a $\:{C}_{\mathrm{max}}$ spike, especially if intolerance appears. Conversely, the male exposure profile is backloaded, reflecting prolonged persistence driven by a longer terminal half-life and greater apparent distribution of CBD and its active metabolite, 7-OH-CBD. Under repeated dosing conditions in mice, this profile may increase the likelihood of accumulation, higher trough concentrations, and slower washout. These findings suggest that preclinical dosing regimens in male mice may benefit from careful consideration of dosing interval and frequency to avoid unintended accumulation that could confound interpretation of efficacy or safety endpoints.

Preclinical studies have reported some sex-dependent differences in CBD exposure, although the available data remain limited and without clear consensus. In rats, oral CBD administration has been associated with a higher C_max_ in females compared with males, while elimination rate constants were not consistently different (Child and Tallon 2022). Also, sex-dependent behavioral responses to CBD have been described. For example, Kaplan et al. (Kaplan et al. 2021) reported significant sex differences in spatial memory performance (Barnes maze) in C57BL/6J mice following intraperitoneal CBD administration, and Ledesma et al. (Ledesma-Corvi et al. 2022) observed sex-dependent antidepressant-like effects in rats after intraperitoneal dosing. Although these studies demonstrate biological sex influences on CBD-related outcomes, the pharmacokinetic mechanisms underlying these differences were not clearly defined. Importantly, the present study provides insights that may help reconcile some of the mixed clinical observations reported in humans. While Zhang et al. (Zhang et al. 2024) observed higher plasma levels of 7-COOH-CBD in women and MacNair et al. (MacNair et al. 2024) also reported greater concentrations of both 7-OH-CBD and 7-COOH-CBD in women, neither study could determine the mechanistic basis for the observed metabolite differences. The present preclinical findings suggest that such differences may arise from sex-dependent distribution and tissue retention of CBD and its metabolites rather than differences in metabolic capacity.

Limitations of the present study include the fact that a single, high IP dose was used. However, the CBD dose (120 mg/kg) and IP route of administration utilized in this study are consistent with extensive preclinical literature in mice, where this dose has been employed to achieve quantifiable systemic exposure and to characterize the pharmacological and behavioral effects of cannabinoids in mice models of epilepsy, neuroinflammation, ethanol consumption and relapse, as well as depression and anxiety (Deiana et al. 2012, Li et al. 2019, Viudez-Martínez et al. 2018, Liu and Burnham 2019). This dosing was specifically chosen to ensure that the primary metabolites, 7-OH-CBD and 7-COOH-CBD, remained above the LLOQ throughout the 24-hour sampling period, allowing for a comprehensive characterization of their pharmacokinetic profiles. Furthermore, the single-dose design permits clear characterization of the terminal phase and associated pharmacokinetic parameters without confounding from accumulation or time-dependent effects. In addition, the IP route was chosen to provide reproducible systemic exposure while minimizing the significant variability and low bioavailability (typically between 6 and 19%) associated with oral administration in mice. This approach allowed for a focused evaluation of sex-dependent differences in the pharmacokinetics of CBD and its metabolites. Given that IP dosing is extravascular and may permit partial first-pass extraction, the conclusion that sex differences are distribution-driven is inferred from plasma data rather than direct measurements of bioavailability. Studies examining tissue concentrations, plasma protein binding, or hepatic/extrahepatic enzyme/transporter expressions were not performed, so the observed effects could not be assessed with respect to specific biological determinants. Future studies employing repeated dosing could be used to assess sex differences in accumulation and steady-state exposure. Coupling such studies with tissue pharmacokinetic measurements (e.g., liver, adipose, brain) would enable direct evaluation of the proposed depot and help identify the anatomical source of the larger Vz/F in males.

## Conclusion

In summary, sex was shown to be associated with a significant difference in the PK of CBD and its metabolites, marked by higher early peaks in female mice and greater persistence in male mice, despite comparable apparent clearance. Therefore, this study demonstrates that sex may be a critical variable influencing CBD disposition in preclinical models and highlights the importance of sex-informed PK study design and interpretation. Collectively, these results provide a mechanistic framework that may help inform future translational and clinical investigations of CBD as its therapeutic use continues to expand.

## Supplementary Information

Supplementary Material 1: Figure 1. Representative chromatograms of blank mouse plasma with no added analytes except D3 internal standards, screened for, (A) CBD; (B) CBD-D3; (C) 7-OH-CBD; (D) 7-OH-CBD-D3; (E) 7-COOH-CBD; and (F) 7-COOH-CBD-D3. Data shown are from a representative C57BL/6J mouse plasma sample and acquired in ESI+ mode following protein precipitation and reversed-phase separation. No endogenous or interfering peaks were observed at the respective analyte retention times.

Supplementary Material 2: Figure 2. Representative chromatograms of CBD and its metabolites with their deuterated internal standard in blank mouse plasma spiked with reference standards. (A) CBD; (B) CBD-D3; (C) 7-OH-CBD; (D) 7-OH-CBD-D3; (E) 7-COOH-CBD; (F) 7-COOH-CBD-D3. Blank mouse plasma was spiked with a reference standard mixture of CBD, 7-OH-CBD, and 7-COOH-CBD at 400 ng/mL and an internal standard mixture at 0.5 ppm, then processed and analyzed by UPLC–MS/MS. Data shown are from a representative C57BL/6J mouse plasma sample acquired in ESI+ mode following protein precipitation and reversed-phase separation. Retention times and monitored mass transitions are annotated above each peak.

Supplementary Material 3.

## Data Availability

All data supporting these findings are contained within the paper.

## Abbreviations

$\:{AUC}_{0-\mathrm{I}\mathrm{N}\mathrm{F}}$: Area under the plasma concentration–time curve from time zero to infinity
$\:{AUC}_{0-24\mathrm{h}}$: Area under the plasma concentration–time curve from time zero to 24 h
CBD: Cannabidiol
CL: Clearance
CL/F: Apparent clearance
$\:{C}_{\mathrm{max}}$: maximum plasma concentration
CYP2C19: Cytochrome P450 2C19
CYP3A4: Cytochrome P450 3A4
FDA: U.S. Food and Drug Administration
GPR55: G protein–coupled receptor 55
IP: Intraperitoneal
LLOQ: Lower limit of quantification
NCA: Non-compartmental analysis
PK: Pharmacokinetics
SD: Standard deviation
t_1/2_: terminal half-life
$\:{t}_{\mathrm{max}}$: time to reach maximum plasma concentration
TRPV1: Transient receptor potential vanilloid type 1
UPLC–MS/MS: Ultra-performance liquid chromatography–tandem mass spectrometry
Vz/F: Apparent volume of distribution
Vd: Volume of distribution
7-OH-CBD: 7-hydroxy-cannabidiol
7-COOH-CBD: 7-carboxy-cannabidiol

## References

[CR1] Premoli M, Aria F, Bonini SA, Maccarinelli G, Gianoncelli A, Pina SD, et al. Cannabidiol: Recent advances and new insights for neuropsychiatric disorders treatment. Life Sci. 2019;224:120–7. 30910646 10.1016/j.lfs.2019.03.053

[CR2] Shahbazi F, Grandi V, Banerjee A, Trant JF. Cannabinoids and Cannabinoid Receptors: The Story so Far. iScience. 2020;23(7):101301. 32629422 10.1016/j.isci.2020.101301 PMC7339067

[CR3] Mechoulam R, Peters M, Murillo-Rodriguez E, Hanuš LO. Cannabidiol – Recent Adv Chem Biodivers. 2007;4(8):1678–92. 10.1002/cbdv.200790147 17712814

[CR4] Stella N. THC and CBD: Similarities and differences between siblings. Neuron. 2023;111(3):302–27. 36638804 10.1016/j.neuron.2022.12.022 PMC9898277

[CR5] Martínez V, Iriondo De-Hond A, Borrelli F, Capasso R, Del Castillo MD, Abalo R. Cannabidiol and Other Non-Psychoactive Cannabinoids for Prevention and Treatment of Gastrointestinal Disorders: Useful Nutraceuticals? Int J Mol Sci. 2020;21(9):3067. 32357565 10.3390/ijms21093067 PMC7246936

[CR6] Oberbarnscheidt T, Miller NS. The Impact of Cannabidiol on Psychiatric and Medical Conditions. J Clin Med Res. 2020;12(7):393–403. 32655732 10.14740/jocmr4159 PMC7331870

[CR7] Zou S, Kumar U. Cannabinoid Receptors and the Endocannabinoid System: Signaling and Function in the Central Nervous System. Int J Mol Sci. 2018;19(3):833. 29533978 10.3390/ijms19030833 PMC5877694

[CR8] Peng J, Fan M, An C, Ni F, Huang W, Luo J. A narrative review of molecular mechanism and therapeutic effect of cannabidiol (CBD). Basic Clin Pharmacol Toxicol. 2022;130(4):439–56. 35083862 10.1111/bcpt.13710

[CR9] Schouten M, Dalle S, Mantini D, Koppo K. Cannabidiol and brain function: current knowledge and future perspectives. Front Pharmacol. 2024;14:1328885. 10.3389/fphar.2023.1328885 10.3389/fphar.2023.1328885 PMC10823027 38288087

[CR10] Miao Y, Zhao F, Guan W. A novel insight into the antidepressant effect of cannabidiol: possible involvement of the 5-HT1A, CB1, GPR55, and PPARγ receptors. Int J Neuropsychopharmacol. 2025;28(2):pyae064. 10.1093/ijnp/pyae064 10.1093/ijnp/pyae064 PMC11878560 39657242

[CR11] De Almeida DL, Devi LA. Diversity of molecular targets and signaling pathways for CBD. Pharmacol Res Perspect. 2020;8(6):e00682 . 10.1002/prp2.682 10.1002/prp2.682 PMC7652785 33169541

[CR12] Filipiuc LE, Ababei DC, Alexa-Stratulat T, Pricope CV, Bild V, Stefanescu R, et al. Major Phytocannabinoids and Their Related Compounds: Should We Only Search for Drugs That Act on Cannabinoid Receptors? Pharmaceutics. 2021;13(11):1823. 34834237 10.3390/pharmaceutics13111823 PMC8625816

[CR13] Dickson K, Janasie C, Willett KL. Cannabinoid Conundrum: A Study of Marijuana and Hemp Legality in the United States. Arizona J Environ Law Policy. 2019;10(20):132–50. 34007734 PMC8127630

[CR14] Park J-Y. Patterns of cannabidiol use among marijuana users in the United States. Prev Med Rep. 2025;50:102985. 39911836 10.1016/j.pmedr.2025.102985 PMC11795096

[CR15] Bhamra SK, Desai A, Imani-Berendjestanki P, Horgan M. The emerging role of cannabidiol (CBD) products; a survey exploring the public’s use and perceptions of CBD. Phytother Res. 2021;35(10):5734–40. 34350641 10.1002/ptr.7232

[CR16] CBD retail In the United States. 2025 [cited 12 March 2026]. Available from: https://www.statista.com/topics/6262/cbd-retail-in-the-united-states/.

[CR17] Nguyen C, Moeller KE, McGuire M, Melton BL. Consumer perception, knowledge, and uses of cannabidiol. Ment Health Clin. 2023;13(5):217–24. 38131055 10.9740/mhc.2023.10.217 PMC10732126

[CR18] Krowartz E-M, Riemerschmid C, Klug SJ, Tanaka LF, Eva H. Beyond the hype - who uses cannabidiol for self-medication – and why: a cross-sectional study in Germany. J Cannabis Res. 2025;7(1):77. 10.1186/s42238-025-00341-4 10.1186/s42238-025-00341-4 PMC12529850 41094600

[CR19] Chesney E, McGuire P, Freeman TP, Strang J, Englund A. Lack of evidence for the effectiveness or safety of over-the-counter cannabidiol products. Therapeutic Adv Psychopharmacol. 2020;10:204512532095499. 10.1177/2045125320954992 PMC7491225 32973998

[CR20] Jazz, Pharmaceuticals. Inc. EPIDIOLEX^®^ [prescribing information]. Carlsbad (CA): Jazz Pharmaceuticals Inc.; 2023. Available from: https://pp.jazzpharma.com/pi/epidiolex.en.USPI.pdf

[CR21] Reddy DS. Therapeutic and clinical foundations of cannabidiol therapy for difficult-to-treat seizures in children and adults with refractory epilepsies. Exp Neurol. 2023;359:114237. 36206806 10.1016/j.expneurol.2022.114237

[CR22] Kaufmann R, Harris Bozer A, Jotte AK, Aqua K, Long-Term, Self-Dosing CBD, Users. Indications, Dosage, and Self-Perceptions on General Health/Symptoms and Drug Use. Med Cannabis Cannabinoids. 2023;6(1):77–88. 37900894 10.1159/000531666 PMC10601936

[CR23] Millar SA, Stone NL, Yates AS, O’Sullivan SE. A Systematic Review on the Pharmacokinetics of Cannabidiol in Humans. Front Pharmacol. 2018;9:1365. 10.3389/fphar.2018.01365 10.3389/fphar.2018.01365 PMC6275223 30534073

[CR24] Martinez Naya N, Kelly J, Corna G, Golino M, Polizio AH, Abbate A, et al. An Overview of Cannabidiol as a Multifunctional Drug: Pharmacokinetics and Cellular Effects. Molecules. 2024;29(2):473. 38257386 10.3390/molecules29020473 PMC10818442

[CR25] Wermer K, Korbacska-Kutasi O, Berkecz R, Csupor D, Ágh N, Sztojkov-Ivanov A et al. Pharmacokinetics of cannabidiol and its two main phase I metabolites in Connemara ponies. Front Veterinary Sci. 2025;12:1599934. 10.3389/fvets.2025.1599934 10.3389/fvets.2025.1599934 PMC12247600 40654508

[CR26] Deiana S, Watanabe A, Yamasaki Y, Amada N, Arthur M, Fleming S, et al. Plasma and brain pharmacokinetic profile of cannabidiol (CBD), cannabidivarine (CBDV), ∆9-tetrahydrocannabivarin (THCV) and cannabigerol (CBG) in rats and mice following oral and intraperitoneal administration and CBD action on obsessive–compulsive behavi. Psychopharmacology. 2012;219(3):859–73. 21796370 10.1007/s00213-011-2415-0

[CR27] Lacerda M, Carona A, Castanheira S, Falcão A, Bicker J, Fortuna A. Pharmacokinetics of Non-Psychotropic Phytocannabinoids. Pharmaceutics. 2025;17(2):236. 40006604 10.3390/pharmaceutics17020236 PMC11858989

[CR28] Hossain KR, Alghalayini A, Valenzuela SM. Current Challenges and Opportunities for Improved Cannabidiol Solubility. Int J Mol Sci. 2023;24(19):14514. 37833962 10.3390/ijms241914514 PMC10572536

[CR29] Bardhi K, Coates S, Watson CJW, Lazarus P. Cannabinoids and drug metabolizing enzymes: potential for drug-drug interactions and implications for drug safety and efficacy. Expert Rev Clin Pharmacol. 2022;15(12):1443–60. 36384377 10.1080/17512433.2022.2148655

[CR30] Beers JL, Fu D, Jackson KD. Cytochrome P450-Catalyzed Metabolism of Cannabidiol to the Active Metabolite 7-Hydroxy-Cannabidiol. Drug Metab Dispos. 2021;49(10):882–91. 34330718 10.1124/dmd.120.000350 PMC11025033

[CR31] Martin BR, Harvey DJ, Paton WD. Biotransformation of cannabidiol in mice. Identification of new acid metabolites. Drug Metab Dispos. 1977;5(3):259–67. 17524

[CR32] Bornheim LM, Correia MA. Selective inactivation of mouse liver cytochrome P-450IIIA by cannabidiol. Mol Pharmacol. 1990;38(3):319–26. 2402224

[CR33] Zhang Q, Melchert PW, Markowitz JS. Pharmacokinetic Variability of Oral Cannabidiol and Its Major Metabolites after Short-Term High-Dose Exposure in Healthy Subjects. Med Cannabis Cannabinoids. 2024;7(1):1–9. 38292071 10.1159/000535726 PMC10824522

[CR34] Ujváry I, Hanuš L. Human Metabolites of Cannabidiol: A Review on Their Formation, Biological Activity, and Relevance in Therapy. Cannabis Cannabinoid Res. 2016;1(1):90–101. 28861484 10.1089/can.2015.0012 PMC5576600

[CR35] Sallam NA, Peterson CS, Baglot SL, Kohro Y, Trang T, Hill MN, et al. Sex Differences in Plasma, Adipose Tissue, and Central Accumulation of Cannabinoids, and Behavioral Effects of Oral Cannabis Consumption in Male and Female C57BL/6 Mice. Int J Neuropsychopharmacol. 2023;26(11):773–83. 37715955 10.1093/ijnp/pyad055 PMC10674081

[CR36] MacNair L, Kulpa J, Hill ML, Eglit GML, Mosesova I, Bonn-Miller MO, et al. Sex Differences in the Pharmacokinetics of Cannabidiol and Metabolites Following Oral Administration of a Cannabidiol-Dominant Cannabis Oil in Healthy Adults. Cannabis Cannabinoid Res. 2024;9(4):e1170–8. 37267269 10.1089/can.2022.0345

[CR37] Waxman DJ, Holloway MG. Sex Differences in the Expression of Hepatic Drug Metabolizing Enzymes. Mol Pharmacol. 2009;76(2):215–28. 19483103 10.1124/mol.109.056705 PMC2713118

[CR38] Aljohmani A, Yildiz D. Biological sex differences in pharmacokinetics and adverse drug reactions. Naunyn-Schmiedeberg’s Archives Pharmacol. 2025;399(3):3285-3301 . 10.1007/s00210-025-04721-8 10.1007/s00210-025-04721-8 PMC12935741 41117965

[CR39] Health UDo, Services H. Food and Drug Administration, Center for Drug Evaluation and Research, CDER. (No Title). 2003.

[CR40] Guideline I. M10 on bioanalytical method validation and study sample analysis. Amsterdam, The Netherlands: European Medicines Agency; 2022.

[CR41] Fashe MM, Miner TA, Collazo VL, Grieco JT, Fallon JK, Jackson KD, et al. Impact of sex and pregnancy on hepatic CYP3A4 expression and activity in a humanized mouse model. Drug Metab Dispos. 2025;53(2):100025. 40023573 10.1016/j.dmd.2024.100025 PMC12105745

[CR42] Sakuma T, Kawasaki Y, Jarukamjorn K, Nemoto N. Sex Differences of Drug-metabolizing Enzyme: Female Predominant Expression of Human and Mouse Cytochrome P450 3A Isoforms. J Health Sci. 2009;55(3):325–37.

[CR43] Wiley JL, Burston JJ. Sex differences in ∆9-tetrahydrocannabinol metabolism and in vivo pharmacology following acute and repeated dosing in adolescent rats. Neurosci Lett. 2014;576:51–5. 24909619 10.1016/j.neulet.2014.05.057 PMC4106361

[CR44] Child RB, Tallon MJ. Cannabidiol (CBD) Dosing: Plasma Pharmacokinetics and Effects on Accumulation in Skeletal Muscle, Liver and Adipose Tissue. Nutrients. 2022;14(10):2101. 10.3390/nu14102101 10.3390/nu14102101 PMC9146469 35631242

[CR45] Kaplan JS, Wagner JK, Reid K, McGuinness F, Arvila S, Brooks M, et al. Cannabidiol Exposure During the Mouse Adolescent Period Is Without Harmful Behavioral Effects on Locomotor Activity, Anxiety, and Spatial Memory. Front Behav Neurosci. 2021;15:711639. 34512286 10.3389/fnbeh.2021.711639 PMC8426900

[CR46] Ledesma-Corvi S, Hernández-Hernández E, García-Fuster MJ. Exploring pharmacological options for adolescent depression: a preclinical evaluation with a sex perspective. Transl Psychiatry. 2022;12(1):220. 35650182 10.1038/s41398-022-01994-y PMC9160287

[CR47] Li CK, Mielnik CA, Ramsey AJ, Ross RA, Burnham WM. The behavioral effects of CBD and THC in mouse models of psychosis. Clin Neurophysiol. 2019;130(8):e119.

[CR48] Viudez-Martínez A, García‐Gutiérrez MS, Navarrón CM, Morales‐Calero MI, Navarrete F, Torres‐Suárez AI, et al. Cannabidiol reduces ethanol consumption, motivation and relapse in mice. Addict Biol. 2018;23(1):154–64. 28194850 10.1111/adb.12495

[CR49] Liu J, Burnham M. The effects of CBD and THC in animal models of depression and anxiety. Clin Neurophysiol. 2019;130(8):e118–9.

