# Supplementary figures and images for: Sex differences in the disposition of cannabidiol and its metabolites in mice
Source: J Cannabis Res. 2026 Apr 2;8:65. doi: 10.1186/s42238-026-00427-7 (PMC13169814; doi:10.1186/s42238-026-00427-7)

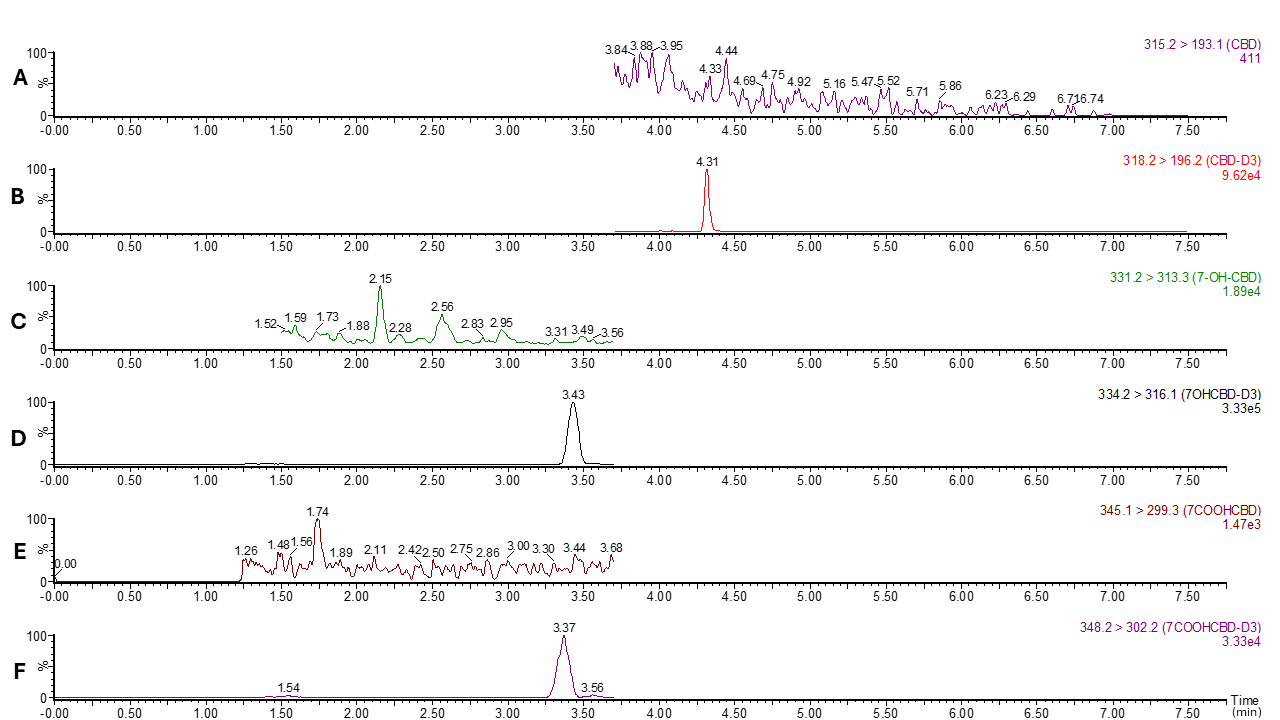

Supplement: Supplementary file 1 — Supplementary Material 1: Figure 1. Representative chromatograms of blank mouse plasma with no added analytes except D3 internal standards, screened for, (A) CBD; (B) CBD-D3; (C) 7-OH-CBD; (D) 7-OH-CBD-D3; (E) 7-COOH-CBD; and (F) 7-COOH-CBD-D3. Data shown are from a representative C57BL/6J mouse plasma sample and acquired in ESI+ mode following protein precipitation and reversed-phase separation. No endogenous or interfering peaks were observed at the respective analyte retention times. [file 42238_2026_427_MOESM1_ESM.tif]

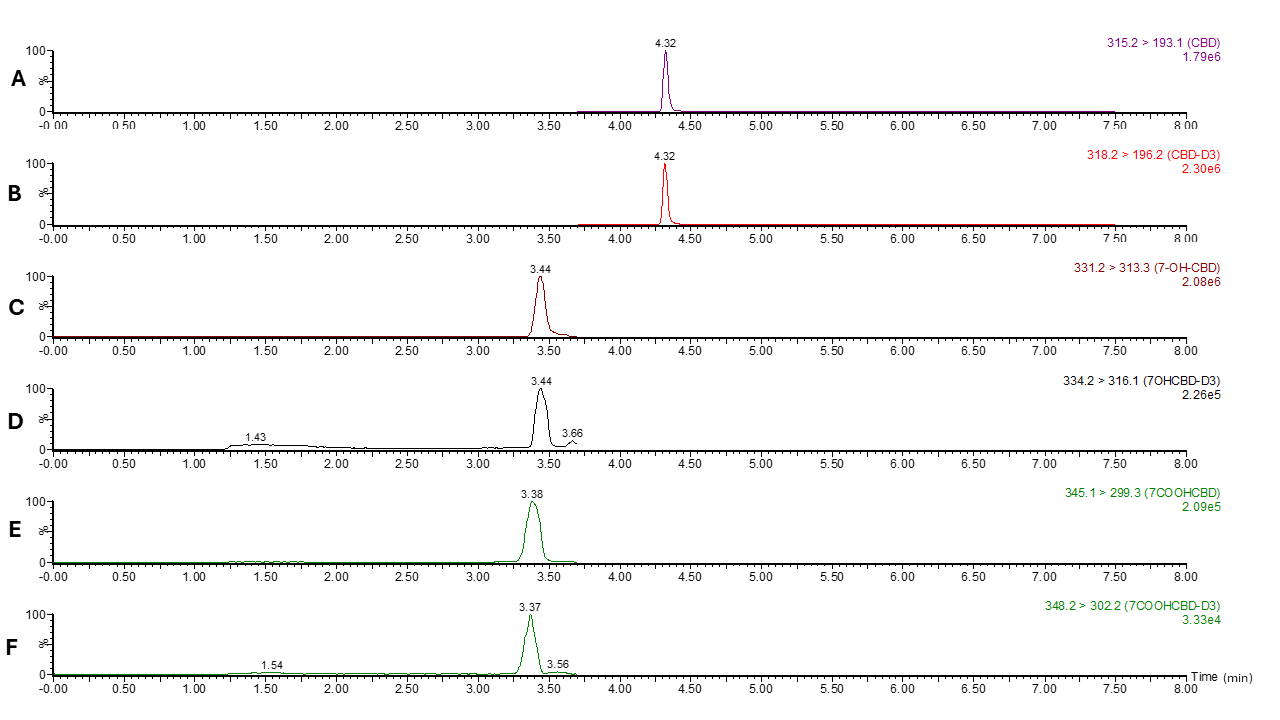

Supplement: Supplementary file 2 — Supplementary Material 2: Figure 2. Representative chromatograms of CBD and its metabolites with their deuterated internal standard in blank mouse plasma spiked with reference standards. (A) CBD; (B) CBD-D3; (C) 7-OH-CBD; (D) 7-OH-CBD-D3; (E) 7-COOH-CBD; (F) 7-COOH-CBD-D3. Blank mouse plasma was spiked with a reference standard mixture of CBD, 7-OH-CBD, and 7-COOH-CBD at 400 ng/mL and an internal standard mixture at 0.5 ppm, then processed and analyzed by UPLC–MS/MS. Data shown are from a representative C57BL/6J mouse plasma sample acquired in ESI+ mode following protein precipitation and reversed-phase separation. Retention times and monitored mass transitions are annotated above each peak. [file 42238_2026_427_MOESM2_ESM.tif]
