# Supplementary material for: Sex differences in the disposition of cannabidiol and its metabolites in mice
Source: J Cannabis Res. 2026 Apr 2;8:65. doi: 10.1186/s42238-026-00427-7 (PMC13169814; doi:10.1186/s42238-026-00427-7)
Supplement: Supplementary file 3 — Supplementary Material 3. [file 42238_2026_427_MOESM3_ESM.docx]

**Supplementary Table 1:** LLOQ validation for CBD, 7-OH-CBD, and 7-COOH-CBD in mouse plasma.

| Analyte | Nominal Concentration LLOQ ^a^ (ng/mL) | Mean ± SD (ng/mL) ^b^ | Accuracy (%) ^c^ | Precision  (CV, %) ^d^ | S/N^e^ |
| --- | --- | --- | --- | --- | --- |
| CBD | 25 | 22.2 ± 1.07 | 89.8 | 4.80 | >10 |
| 7-OH-CBD | 50 | 54.5 ± 1.99 | 109 | 3.70 | >10 |
| 7-COOH-CBD | 50 | 50.3 ± 3.14 | 101 | 6.20 | >10 |

^a^ LLOQ, lower limit of quantification.

^b^ LLOQ values were determined from five replicate measurements.

^c^ Accuracy (%), mean measured concentration/nominal concentration X 100.

^d^ Precision (CV, %), standard deviation/mean measured concentration X 100.

^e^ S/N, signal-to-noise ratio.

**Supplementary Table 2**. Intra-day and inter-day accuracy and precision of UPLC-MS/MS method used for quantifying CBD, 7-OH-CBD and 7-COOH-CBD in mouse plasma.

| Analyte | QC Level ^a^ | Nominal Concentration (ng/mL) | Intra-Day (*n* = 5) | | | Inter-Day (*n* = 15) | | |
| --- | --- | --- | --- | --- | --- | --- | --- | --- |
|  |  |  | **Mean ± SD (ng/mL)** | **Accuracy (%) ^b^** | **Precision**  **(CV, %) ^c^** | **Mean ± SD**  **(ng/mL)** | **Accuracy (%)** | **Precision**  **(CV, %)** |
| CBD | LQC | 200 | 196 ± 4.47 | 98.0 | 2.28 | 193 ± 5.17 | 96.5 | 2.68 |
|  | MQC | 6400 | 5855 ± 105 | 91.4 | 1.79 | 5842 ± 83 | 91.3 | 1.42 |
|  | HQC | 12800 | 11847 ± 154 | 92.6 | 1.30 | 12028 ± 277 | 94.0 | 2.30 |
| 7-OH-CBD | LQC | 200 | 193 ± 2.27 | 96.7 | 1.17 | 192 ± 4.73 | 95.9 | 2.47 |
|  | MQC | 6400 | 5577 ± 234 | 87.1 | 4.20 | 5 706 ± 264 | 89.1 | 4.63 |
|  | HQC | 12800 | 11731 ± 305 | 91.7 | 2.60 | 11851 ± 287 | 92.6 | 2.43 |
| 7-COOH-CBD | LQC | 200 | 184 ± 3.50 | 92.1 | 1.90 | 188 ± 5.99 | 94.1 | 3.18 |
|  | MQC | 6 400 | 5 575 ± 38.4 | 87.1 | 0.69 | 5 611 ± 84.9 | 87.7 | 1.51 |
|  | HQC | 12 800 | 12 140 ± 305 | 94.8 | 2.51 | 12 074 ± 233 | 94.3 | 1.93 |

^a^QC, quality control; LQC, low quality control; MQC, medium quality control; HQC, high quality control.

^b^ Accuracy (%), mean measured concentration/nominal concentration X 100.

^c^ Precision (CV, %), standard deviation/mean measured concentration X 100.
